# Ceftaroline Exhibits Promising *In Vitro* Activity Against Methicillin-Resistant *Staphylococcus aureus* Clinical Isolates From Alexandria, Egypt

**DOI:** 10.1155/ijm/4558662

**Published:** 2025-05-19

**Authors:** Hussien H. Sallam, Asmaa A. Ramadan, Nancy M. Attia, Amira ElBaradei, Sherine M. Shawky, Mohammed A. El-Kholy

**Affiliations:** ^1^Department of Microbiology, Medical Research Institute, Alexandria University, Alexandria, Egypt; ^2^Department of Microbiology and Biotechnology, Clinical and Biological Sciences Division, College of Pharmacy, Arab Academy for Science, Technology and Maritime Transport (AASTMT), Alexandria, Egypt; ^3^Ministry of Health and Population, Alexandria, Egypt; ^4^Department of Microbiology and Immunology, College of Medicine and Health Sciences, Sultan Qaboos University, Muscat, Oman

**Keywords:** ceftaroline, Egypt, methicillin-resistant *Staphylococcus aureus* (MRSA), resistance, sensitivity, *spa* typing

## Abstract

**Introduction:** Ceftaroline is a fifth-generation cephalosporin that was recently introduced into the Egyptian market for the treatment of methicillin-resistant *Staphylococcus aureus* (MRSA) infections. However, limited data are available regarding the susceptibility of MRSA isolates in Egypt to this antibacterial agent. This study aimed to determine the susceptibility of MRSA strains recovered from different clinical samples to ceftaroline and to investigate the prevalence of the *mec*A and *mec*C resistance genes.

**Methods:** A total of 412 MRSA isolates were selected from 520 *Staphylococcus aureus* (*S. aureus*) samples. Identification and antibiotic susceptibility testing were performed using the VITEK-2 compact system. Molecular identification of the *nuc* gene, encoding nuclease enzyme, a species-specific marker for *S. aureus*, and the *mec*A and *mec*C genes associated with methicillin resistance was performed using the polymerase chain reaction (PCR) technique. Moreover, the *in vitro* activity of ceftaroline was explored using the disc diffusion method, and its minimum inhibitory concentration (MIC) was determined according to the Clinical Laboratory Standards Institute (CLSI) criteria. Staphylococcal Protein A (*spa*) typing was carried out for ceftaroline nonsusceptible strains as determined by MIC.

**Results:** Most isolates were recovered from skin and soft tissue infections. Of the 412 clinical isolates, 407 (98.7%) were susceptible to ceftaroline, with an MIC of ≤ 1 mg/L, while five isolates (1.3%) showed a susceptible dose–dependent (SDD) profile with MIC values of 2–4 *μ*g/mL. No isolates were resistant to ceftaroline. All isolates carried the *nuc* gene, 94% harbored *mecA*, while *mecC* was undetected. Of the five SDD isolates, three were identified as *spa* type t037, corresponding to ST-239, ST-240, or ST-241 by multilocus sequence type (MLST), whereas the two remaining isolates were untypeable.

**Conclusions:** From various clinical samples, ceftaroline demonstrated excellent *in vitro* activity against MRSA strains, positioning it as a promising therapeutic option for managing MRSA infections in Egypt.

## 1. Introduction

Antimicrobial resistance remains a challenging threat worldwide, affecting both developed and developing countries. A high number of annual morbidities and mortalities is attributed to the antimicrobial resistance problem [1]. Methicillin-resistant *Staphylococcus aureus* (MRSA) plays the most significant role in this situation, causing different therapeutically challenging infections in hospital settings and the community.

In Egypt, MRSA represents a major public health concern, particularly within healthcare environments, in addition to being a leading cause of severe life-threatening infections [2]. The prevalence of multidrug-resistant (MDR) MRSA strains complicates treatment, thus making these infections more challenging to address [3]. Furthermore, the frequent existence of both virulence factors and resistance genes in MRSA isolates enhances the pathogen's capacity to resist standard antimicrobial treatments [4, 5]. These findings underscore the critical need for robust infection control strategies and antibiotic stewardship programs to reduce the impact of MRSA in Egypt [2].

MRSA was first reported in 1961 in England [6]. MRSA possesses an altered penicillin-binding protein called PBP2a, encoded by the *mecA* gene, which reduces the affinity for *β*-lactam antibiotics, serving as the primary genetic determinant to resistance against all *β*-lactam antibiotics in MRSA [7]. In 2011, a homologous gene, *mecC*, was identified for the first time in MRSA isolates from dairy cattle and humans in the United Kingdom and Denmark. Initially, it was known as *mecALGA251* before being renamed *mecC*. This gene encodes PBP2c, another low-affinity penicillin-binding protein that confers methicillin resistance. Although *mecC* remains less prevalent and has primarily been reported in European animal and human isolates, its potential emergence in other regions, including Egypt, necessitates its inclusion in MRSA detection efforts [8, 9].

A constant search was necessary to build a collection of effective agents to treat MRSA infections. Glycopeptides, represented by vancomycin, were considered the cornerstone for effective anti-MRSA therapy. Nonetheless, treatment failures with vancomycin in critically ill patients have mainly been reported to be caused by suboptimal therapeutic levels or high minimum inhibitory concentration (MIC) values. Fortunately, several agents have become available for treating MRSA, including linezolid, daptomycin, telavancin, and ceftaroline [7, 10].

Ceftaroline is a fifth-generation cephalosporin demonstrating broad-spectrum bactericidal activity against MRSA, VISA, heterogeneous VISA (hVISA), and VRSA [11]. Several studies have demonstrated that ceftaroline is well tolerated by patients and is equally effective as vancomycin, daptomycin, and linezolid in eradicating MRSA. Ceftaroline fosamil, the prodrug of ceftaroline, was approved by the US Food and Drug Administration (FDA) in 2010 for treating complicated skin and soft tissue infections caused by MRSA infections as well as community-acquired bacterial pneumonia and acute bacterial/skin and skin structure infections [12]. Ceftaroline was recently introduced to the Egyptian market in November 2020, providing a valuable addition to the limited arsenal of effective antibiotics against MRSA infections.

The enhanced bactericidal activity of ceftaroline against MRSA could be related to its high binding ability to penicillin-binding proteins, specifically to an allosteric site of PBP2a close to the transpeptidase domain [13, 14]. Resistance to ceftaroline is uncommon; however, several studies have reported decreased susceptibility of MRSA to ceftaroline in sporadic cases. This resistance may be due to the mutation within the PBP2a protein, particularly outside the penicillin-binding domain [12]. Notably, resistance was occasionally observed even before ceftaroline was introduced. Data regarding the use of ceftaroline for the treatment of MRSA bacteremia are limited to small retrospective case series [15].

Several genotyping methods have been used to examine *S. aureus* clonal relatedness, as well as the epidemiology of the infection [16]. *spa* typing is one of the most widely used methods for characterizing MRSA. It involves sequencing the repeat region of the *spa* gene and is commonly employed for tracking transmission and investigating outbreaks [17].

Consequently, the main objectives of this study were to provide data regarding the antibiotic susceptibility profile and to assess the *in vitro* activity of ceftaroline against MRSA isolates, which are collected from various clinical samples. The prevalence of *mecA* and *mecC* genes would also be investigated.

## 2. Methods

### 2.1. Bacterial Isolates

In the current study, 412 MRSA isolates were selected from 520 S*. aureus* clinical samples. The isolates were collected from various infections, including skin and soft tissue, surgical sites, mostly orthopedic surgeries, respiratory tract, and bloodstream, among other infections. Samples were collected from different healthcare settings in Alexandria, Egypt, from June 2022, to April 2023.

### 2.2. Identification and Antibiotic Susceptibility Testing

All samples were inoculated on blood agar plates, and *Staphylococcal* isolates were identified by their colonial appearance (size, shape, consistency, pigmentation, and hemolysis). The colonies that were suspected as staphylococci were Gram-stained for their microscopical appearance (Gram-positive cocci in grape-like clusters), and the catalase test was performed.

Identification to the species level and antibiotic susceptibility testing for all the isolates were carried out by the VITEK 2 compact system (BioMérieux, France), using GP identification cards and AST-P592, respectively. The following antibiotics were tested: cefoxitin screening (confirmation of MRSA), benzylpenicillin, ampicillin, oxacillin, imipenem, gentamicin, ciprofloxacin, moxifloxacin, inducible clindamycin resistance, erythromycin, clindamycin, linezolid, teicoplanin, vancomycin, tetracycline, tigecycline, fusidic acid, rifampin, and trimethoprim/sulfamethoxazole. Molecular confirmation of *S. aureus* species was achieved by detecting the *nuc* gene, which encodes a thermostable nuclease enzyme specific to *S. aureus*, acting as a dependable marker for species identification.

### 2.3. *In Vitro* Activity of Ceftaroline Against MRSA

Ceftaroline antimicrobial susceptibility was determined by disk diffusion method using the ceftaroline antibiotic disc (30 *μ*g) (Oxoid, United Kingdom). Results were interpreted as sensitive ≥ 25 mm, susceptible dose–dependent (SDD) 20–24 mm, or resistant ≤ 19 mm. Moreover, ceftaroline MIC was determined as sensitive: ≤ 1 *μ*g/mL, SDD: 2–4 *μ*g/mL, or resistant: ≥ 8 *μ*g/mL. ATCC strains of *S. aureus* (ATCC 25923 and ATCC 43300) were used as controls. Interpretation of results was conducted according to the CLSI guidelines (M100) with respect to ceftaroline breakpoints [18].

### 2.4. Molecular Characterization of MRSA Isolates

#### 2.4.1. DNA Extraction and Amplification of *nuc*, *mecA,* and *mecC* Genes

DNA was extracted from MRSA isolates by boiling method [19]. Specific primers (Table.  1) were utilized to amplify *nuc*, *mecA*, and *mecC* genes by PCR using MyTaq HS Red Mix (Bioline, United Kingdom), following the manufacturer's instructions. Thermal cycling conditions of the PCR were as follows: single cycle as initial denaturation (2 min at 95°C), followed by 30 cycles: denaturation (30 sec at 95°C), annealing (15 sec at 55°C for *mecA* and *nuc* and 50°C for *mecC*), and extension (30 sec at 72°C) followed by a single cycle of final extension (5 min 72°C). Amplified DNA fragments were subjected to agarose gel electrophoresis (2%) followed by gel examination under a UV trans-illuminator at 254 nm to determine the sizes of the separated bands relative to the loaded 50-bp DNA ladder (GeneDirex, Taiwan).

#### 2.4.2. *spa* Typing of Ceftaroline Nonsusceptible MRSA Isolates

The *spa* gene hypervariable region was amplified using MyTaq HS Red Mix (Bioline, United Kingdom) according to the manufacturer's instructions and using the aforementioned specific primers: 1095F (5 = -AGACGATCCTTCGGTGAGC) and 1517R (5 = -GCTTTTGCAATGT CATTTACTG). Thermal cycling conditions of the PCR were as follows: single cycle as initial denaturation (2 min at 95°C), followed by 30 cycles: denaturation (30 sec at 95°C), annealing (15 sec at 55°C), and extension (30 sec at 72°C) followed by a single cycle of final extension (5 min 72°C) [23]. The PCR product was purified from unincorporated primers and dNTPs using the Montage PCR Clean up kit (Millipore, WI). The forward and reverse strands of the purified PCR product were sequenced using BigDye Terminator sequencing kit v3.1 according to the manufacturer's protocol (Applied Biosystems, United States). Sequencing products were resolved on an Applied Biosystems model 3730XL automated DNA sequencing system (Applied Biosystems, United States). The obtained gene sequences were analyzed, and *spa* types were assigned using the Ridom *Spa*Server database (http://spa.ridom.de).

## 3. Results

### 3.1. Distribution of Clinical Isolates and Patterns of Antibiotic Sensitivity Testing

A total of 412 MRSA isolates were collected in this study. The majority of the samples were obtained from males (238 isolates, 57.77%), while the remaining 174 isolates (42.23%) were obtained from females. Isolates were retrieved from a variety of clinical sample types, with skin and soft tissue samples being the most common (163 isolates, 39.56%), followed by bone and orthopedic lesions (82 isolates, 19.9%) and surgical site infections (71 isolates, 17.23%). (Table 2).

**Figure 1:**
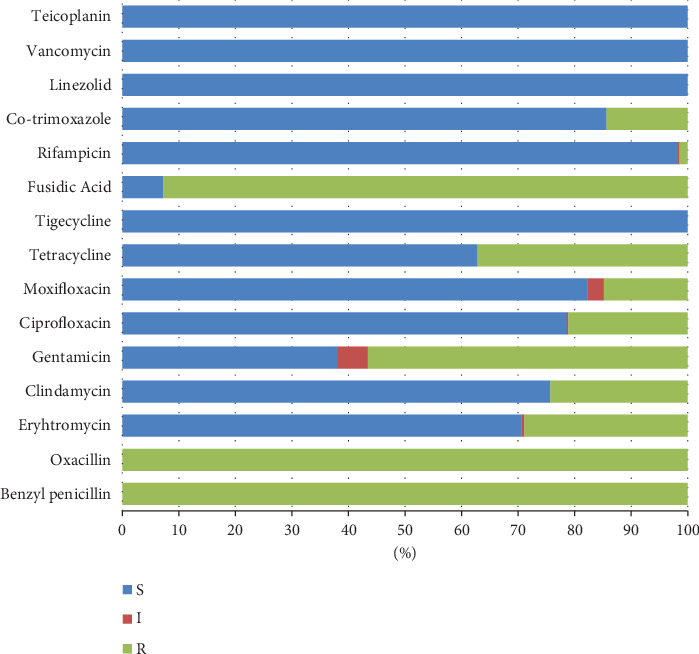
Antibiotic sensitivity levels of MRSA clinical isolates.

**Figure 2:**
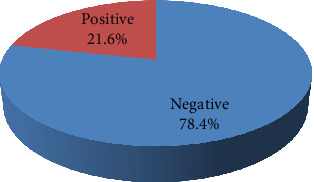
Inducible clindamycin resistance phenotype among MRSA clinical isolates.

Methicillin resistance was confirmed in all the isolates included in the present study by positive cefoxitin screening using a VITEK AST-P592 card. All the isolates were sensitive to vancomycin, teicoplanin, tigecycline, and linezolid, whereas 92.72% of the isolates showed fusidic acid resistance. Variable resistance patterns were observed for the rest of the tested antibiotics; isolates were resistant to gentamicin, tetracycline, erythromycin, clindamycin, ciprofloxacin, moxifloxacin, co-trimoxazole, and rifampicin with percentages of 56.55, 37.14, 28.88, 24.27, 21.12, 14.81, 14.32, and 1.46, respectively. (Figure 1). The incidence of inducible clindamycin resistance was 21.6% (89/412) among the tested isolates. (Figure 2).

The *nuc* gene was present in all MRSA isolates (100%), while the *mecA* gene was detected in 387/412 (94%). On the contrary, no *mecC* gene could be detected in all tested isolates.

### 3.2. *In Vitro* Activity of Ceftaroline Against MRSA Clinical Isolates

#### 3.2.1. Disc Diffusion Method

Similarly, the disk diffusion method did not identify any ceftaroline-resistant isolates. Most ceftaroline-susceptible isolates 121/200 (60.5%) showed inhibition zones ranging between 27 and 30 mm. Among all isolates, only 12 showed SDD patterns. The overall susceptibility of ceftaroline was 94%, according to disk diffusion (Table 3).

#### 3.2.2. Ceftaroline Susceptibility by Determining MIC Values

According to the obtained MIC results, no ceftaroline-resistant isolates were observed, and the overall susceptibility of ceftaroline was 98.79% (407/412). The majority of ceftaroline susceptible isolates 318/407 (78.13%) demonstrated a MIC range of 0.25–0.5 *μ*g/mL. Among all isolates, only five isolates showed an SDD pattern (Table 4).

### 3.3. *spa* Typing Results

*spa* typing for three MRSA clinical isolates was carried out using the Ridom Spa Server database (https://www.spaserver.ridom.de/) and Ridom SeqSphere + software version 7.6.1 (Ridom, Munster, Germany) for *spa* sequence analysis [24, 25]. The three isolates were typed successfully as t037 with identical repeats (15-12-16-02-25-17-24) in one cluster. The reliability of these three isolates was excellent, with a rate of 120. According to the Ridom spa server database (https://spa.ridom.de/spatypes.shtml), the t037 spa type corresponded to either ST-239, ST-240, or ST-241 defined by MLST (multilocus sequencing type) (Table 5). The remaining two SDD isolates could not be typed using this method.

## 4. Discussion

MRSA is a hazardous Gram-positive bacteria representing a significant threat to global health. On a global scale, MRSA strains are becoming more common among *S. aureus* infections. Additionally, MRSA strains have developed resistance to most antibiotics, including *β*-lactams [26, 27]. Globally, MRSA isolates comprise nearly 40% of all *S. aureus* isolates [28]. A previously published systematic analysis revealed that MRSA contributed to over 100,000 deaths and 3.5 million disability-adjusted life years (DALYs) globally. [29]. MRSA infections are associated with more severe clinical outcomes in comparison to methicillin-sensitive *S. aureus* (MSSA), leading to longer hospital stays, higher use of hospital resources, and increased treatment expenses [30]. In Egypt, a recently published systematic review with meta-analysis on MRSA comprised data from 64 studies, including a total of 7,171 clinical isolates that were collected from six regions: Cairo, Mansoura, Zagazig, Alexandria, Assiut, and Tanta. The review reported that the overall prevalence of MRSA among *S. aureus* clinical isolates in Egypt was remarkably high, averaging 63%. The high prevalence highlights the widespread nature of MRSA in Egypt, which is likely influenced by inadequate infection control programs, limited resources, and antibiotic misuse. [2].

Ceftaroline fosamil, the prodrug of ceftaroline, has a strong affinity for penicillin-binding protein (PBP2a), and accounts for its exceptional efficacy against MRSA. Furthermore, ceftaroline has been used clinically to treat MRSA-associated pneumonia and severe MRSA infections like bacteremia and infective endocarditis [31, 32]. As ceftaroline was recently launched in the Egyptian market in 2020, the goal of this study was to assess the drug's *in vitro* activity and susceptibility against MRSA isolates.

The study at hand showed that the prevalence of MRSA isolates among the collected *S. aureus* isolates was 79.23%. The current results align with the aforementioned high rates of MRSA infections in Alexandria and generally in Egypt [2]. In addition, the findings of the present study agree with other Egyptian studies such as Alfeky et al. (2022) who reported high MRSA rates, which accounted for almost 80% of all *S. aureus* isolates [33]. Falagas et al. also mentioned that in Egypt, the spread of MRSA was 45% and 52% between 2003 and 2005, respectively, but it was as high as 82% among cancer patients [34]. A comparison between earlier and recent studies suggests that MRSA rates have remained constantly high over time, and hence highlighting a failure to control its spread despite the growing awareness. These alarming rates underscore the urgent need for effective strategies to combat MRSA infections in Egypt.

Several interconnected factors are likely to contribute to Egypt's high MRSA prevalence. The widespread antibiotic misuse and overuse, driven by easy over-the-counter access and self-medication, create strong selective pressure for resistant strains to emerge and dominate. Inadequate infection control measures, including gaps in surveillance, poor hand hygiene, and insufficient patient isolation protocols, can further accelerate MRSA transmission in both community and healthcare settings. A combination of these factors can foster an environment conducive to MRSA persistence and spread [35–38].

An awareness campaign in Alexandria, Egypt, has significantly improved public knowledge and behavior regarding antibiotic misuse, which contributed to reducing self-medication for common colds from 22% to 7%. This campaign also led to a 1.6-fold increase in those seeking healthcare professionals' advice before starting antibiotics and a 1.3-fold rise in adherence to completing prescribed courses. Despite these improvements, misconceptions about when to take antibiotics persists, emphasizing the need to improve public education on the dangers of antibiotic misuse may help reduce the selective pressure contributing to MRSA prevalence [39]. Addressing these ongoing public health challenges requires the urgent implementation of targeted strategies, including antimicrobial stewardship programs, routine MRSA screening in high-risk hospital units, and more stringent enforcement of infection prevention protocols.

Different samples were collected from various origins of infections, including skin and soft tissue samples in 79 (48.5%), followed by wounds and/or surgical site infections in 37 (18.5%), and pulmonary specimens in 30 (15%). Bloodstream infections also accounted for 5% of the isolates.

The current findings are similar to a related study conducted in Kuwait, which examined 453 S*. aureus* isolates—265 MRSA and 188 MSSA isolates—obtained from 12 hospitals. The respiratory tract (11.9%), blood (5.9%), anterior nares (20.3%), SSTIs (39.3%), and other sources (15.4%) were the origins of the isolates [40]. Furthermore, the present results also aligned with a recent study in Alexandria, wherein most MRSA isolates were acquired from wound swabs and aspirated pus [41]. On the other hand, other studies found that most MRSA clinical isolates were recovered from blood samples [42, 43].

In the present study, all the MRSA isolates were sensitive to linezolid, tigecycline, teicoplanin, and vancomycin. These findings are in agreement with previous Egyptian studies that reported 100% susceptibility of the studied isolates to vancomycin and linezolid [42, 44]. Furthermore, other reports demonstrated comparable susceptibilities to linezolid [45, 46]. In this study, inducible clindamycin resistance was detected in 22% of the isolates, which aligns with a recently published analyzing the prevalence rates of inducible clindamycin resistance among *S. aureus* isolates in Africa. The overall prevalence was reported as 19.8%, with rates ranging from 2.9% to 44.0%. Egypt recorded the highest overall prevalence at 44%, followed by Libya (35.8%) and Uganda (33.3%). Additionally, resistance was found to be more common in MRSA isolates, with rates ranging from 3.6% to 77.8%. In addition, Egypt showed the highest rate, followed by Nigeria (75%) and Libya (66.2%) [47]. Hence, the current findings underscore the need for routine screening, prudent clindamycin use, and molecular characterization of resistance genes in order to prevent further spread of inducible clindamycin-resistant *S. aureus*, particularly in high-burden regions like Egypt.

The current study demonstrated a relatively low moxifloxacin resistance rate of 14.81% among MRSA isolates. Multiple studies have documented moxifloxacin resistance rates [48–51]. When compared to other studies in Egypt across different times, the highest reported resistance was observed by Alseqely et al. [52], who reported a striking 69% resistance rate among 72 clinical MRSA isolates collected in 2015. This rate is notably higher than that of the present study, even though both studies were conducted in the same city—Alexandria—but at different periods of time and with different total numbers of isolates. Additionally, Alseqely et al.'s samples were collected from a single hospital site, which may reflect a localized resistance pattern driven by specific hospital practices or antibiotic usage trends. In contrast, isolates included in the current study were obtained from a diverse range of patients across multiple healthcare facilities throughout Alexandria, representing a broader picture of the city's resistance landscape.

In contrast, a study conducted in another Egyptian city—Zagazig—between 2015 and 2018 documented a significantly lower resistance rate of 6.3%, which is lower than our findings [53]. A study in three university hospitals in Upper Egypt reported a 40% resistance rate among MRSA isolates from hospital-acquired pneumonia cases [54]. Additionally, a study published in 2007 found a 30.8% resistance rate among *S. aureus* isolates from Egyptian cancer patients, which remains higher than our findings [55]. This suggests regional variation or potential differences in the sample type and infection control practices. Earlier studies also showed varying resistance rates.

Using two methods for susceptibility testing MIC evaluator strips and disc diffusion, no ceftaroline resistance was detected in the present study. By comparing the results of the two methods, no discrepancy in results was found. The current results agree with a similar study investigating ceftaroline's antibacterial activity against clinical isolates of methicillin-susceptible and -resistant *S. aureus* in Kuwaiti hospitals. The study reported that ceftaroline demonstrated good *in vitro* activity against both MSSA and MRSA, suggesting that it may be a valuable substitute for vancomycin in the treatment of MRSA infections [40].

Findings from the ATLAS Program's 6 years (2012–2017) claimed that the United States had the highest MRSA susceptibility to ceftaroline, followed by Asia-Pacific, Europe, Africa/West Asia, South America, and South America. Moreover, the SENTRY program found that MRSA and MSSA were 100% and 91.6% susceptible to ceftaroline, respectively [56].

The detection of the *nuc* gene in all study isolates is particularly significant as it produces the thermostable nuclease enzyme. This enzyme breaks down host cell DNA and RNA, causing severe damage to host tissues and aiding the spread of the pathogen, partly by evading neutrophil extracellular traps as well as suppressing biofilm formation. Its presence further confirmed the identification of our isolates as *S. aureus*, since *nuc* is recognized as a reliable marker for detecting *S. aureus* [57–59]. On the other hand, some *S. aureus* strains may have undetectable *nuc* genes. Studies have shown varying rates of *nuc*-negative *S. aureus* isolates. The absence or undetectability of the *nuc* gene can result from genetic variations, as *S. aureus* strains can have diverse genetic makeup, leading to differences in the *nuc* gene sequence or its complete absence. This highlights that reliance on just one species-specific gene for molecular MRSA screening can result in incorrect identification [60, 61].

The cefoxitin screening provided in the VITEK AST-P592 card verified that all 412 strains were methicillin-resistant. However, PCR failed to detect the *mecA* gene in about 6% of the isolates. A Similar finding was also reported in an Egyptian study in 2017 by Rania et al. who investigated methicillin resistance among 600 MRSA and MR-CoNS isolates from two Egyptian hospitals, revealing 5.5% that tested negative for *mec*A by PCR but remained resistant to methicillin through both disc diffusion and VITEK II testing, suggesting the potential involvement of alternative resistance mechanisms [62]. A recent study in Egypt in 2023 stated that 17.8% of *S. aureus* clinical isolates were phenotypically methicillin resistant but did not harbor *mec*A gene [42]. Other several investigations also indicate that some resistant isolates lack *mec*A, although gene identification has long been thought to be the gold standard [63, 64].

This result could be explained as a false negative PCR reaction brought on by a point mutation, deletion, or the presence of PCR inhibitors in the *mec*A gene. Ba et al. demonstrated unequivocally that MRSA's beta-lactam resistance could be caused by mechanisms other than the presence of the *mecA* gene and that molecular techniques alone are insufficient for the accurate characterization of MRSA isolates [65].

Both *mecA* and *mecC* genes only have 70% DNA identity in common. The *mecC* gene was first identified in MRSA isolates from a tank milk sample in southwest England [66]. Following this discovery, it has been frequently detected in livestock and wild animals [67]. However, its occurrence in human isolates remains low, with most reports originating from Europe. The highest prevalence of *mec*C gene among MRSA isolates has been observed in Europe, mainly in the United Kingdom and Denmark [8, 68–75]. In the current study, *mecC* was not detected in any of the PCR-tested MRSA isolates. This finding aligns with several previous studies globally, which also reported the absence of the *mecC* gene in methicillin-resistant staphylococci isolates from human samples [76–79].

Limited information is available on the prevalence of MRSA harboring the *mec*C gene in Egypt, and its frequency was assessed in this study using the PCR method. Notably, none of the examined MRSA isolates was found to carry the *mec*C gene.

Previous studies in Egypt consistently reported the absence of the *mec*C gene in MRSA clinical isolates, even among those lacking the *mec*A gene. One study found the *mec*C gene to be completely absent in MRSA and methicillin-resistant coagulase-negative Staphylococci (MR-CoNS) isolates from hospital-acquired infections [42]. Another study in 2017 detected no *mec*C gene in 34 MRSA isolates from 1300 swabs collected from environmental surfaces, patients, and dental healthcare personnel across six wards in an Egyptian dental clinic [80]. Additionally, a study screening 600 methicillin-resistant staphylococcal isolates (520 MRSA and 80 MR-CoNS) from two Egyptian university hospitals, including 150 discrepant isolates with mismatched cefoxitin and oxacillin susceptibility patterns, also found no *mec*C-positive isolates [62]. On the contrary, the *mecC* gene was first identified in Egypt by Shebl et al., who detected it in three MRSA isolates, accounting for 6% of the total PCR-tested samples. This study took place in Egypt's largest university hospital, which serves a diverse patient population, including individuals from various rural and urban areas [43].

The discrepancy in detection of *mec*A and *mec*C genes may result from genetic mutations in the PBP encoding genes affecting primer-binding regions [81]. Moreover, the presence of PCR inhibitors, or suboptimal PCR conditions, could further contribute to these false negatives [64]. Additionally, the presence of hyper-*β*-lactamase-producing strains, known as borderline oxacillin-resistant *S. aureus* (BORSA), exhibits low-level resistance to oxacillin [82]. These strains do not carry the modified PBP2a protein, which is typically encoded by the *mec*A or *mec*C genes [83]. Furthermore, the potential existence of MRSA strains carrying *mec* gene homologs or other emerging variants should be considered in both diagnostic and infection control strategies. Overlooking these variants may lead to false-negative results, adverse clinical outcomes, and suboptimal infection control measures, further complicating efforts to manage MRSA infections [67].

Given these complexities, the absence of *mec*A and *mec*C genes cannot reliably rule out MRSA [68]. Combining phenotypic and genotypic methods may provide a more reliable approach for detecting MRSA strains, ensuring a clearer understanding of resistance patterns and minimizing the risk of false-negative results [26].

The spa t037 is considered the most prevalent in Africa [84]. In a Kenyan investigation of *S. aureus* carriage by inpatients in a public hospital, only 6 out of 86 (7%) isolates of the bacteria were MRSA, and they were all members of the same clone (MLST ST239; *spa* type t037) [85]. According to another study from South Africa, the most prevalent spa type was t037 [86]. This is consistent with an analysis carried out in 2018 of studies from 2007 onward from Europe, Asia, America, Australia, and Africa, including 18 studies from Africa, which revealed that the most common spa type was t037 [84]. Thus, these data support our finding of the three isolates of *spa* typing.

In conclusion, the current study highlighted the high prevalence of MRSA in Egypt, with ceftaroline demonstrating susceptibility rates comparable to other key antibiotics such as vancomycin, linezolid, and teicoplanin. No *mecC* gene was detected in this study, which is consistent with other research indicating its absence among clinical MRSA isolates in Egypt. To the authors' best knowledge, this is the first Egyptian study to evaluate the *in vitro* activity of ceftaroline against a large collection of MRSA clinical isolates.

Given the rising challenge of antimicrobial resistance, national healthcare authorities are encouraged to prioritize judicious broader access to ceftaroline particularly in hospitals managing severe MRSA infections, where traditional alternatives like vancomycin may be less effective or associated with toxicity. Incorporating ceftaroline into antimicrobial stewardship programs is essential to ensure its appropriateness, evidence-based use and to mitigate the risk of resistance development. Furthermore, expanding clinician awareness and ensuring cost-effective availability through governmental support and strategic procurement policies could optimize ceftaroline's role in managing resistant infections across Egypt, offering a valuable addition to the limited arsenal of effective anti-MRSA therapies.

## Figures and Tables

**Table 1:** Primer names, sequences, melting temperatures (Tm), and corresponding amplicon sizes.

**Primer**	**Nucleotide sequence (5′ to 3′)**	**Tm (°C)**	**Amplicon size (bp)**	**Ref.**
*mecA*	Forward: AAAATCGATGGTAAAGGTTGGC	55	533 bp	[20]
	Reverse: AGTTCTGCAGTACCGGATTTGC			

*mecC*	Forward: GAAAAAAAGGCTTAGAACGCCTC	50	138 bp	[21]
	Reverse: GAAGATCTTTTCCGTTTTCAGC			

*nuc*	Forward: GCGATTGATGGTGATACGGTT	55	279 bp	[22]
	Reverse: AGCCAAGCCTTGACGAACTAAAGC			

**Table 2:** Distribution of sample types by source of infection.

**Source of infection**	**Number (** **N** ** = 412)**	**%**
Skin and soft tissue	163	39.56
Surgical site infection	71	17.23
Bone and orthopedic lesions	82	19.90
Sterile body fluids	19	4.61
Respiratory tract	42	10.19
Blood	27	6.55
Others	8	1.94

**Table 3:** *In vitro* susceptibility of MRSA isolates to ceftaroline by disc diffusion method.

**Inhibition zone (mm)**	**Interpretation according to CLSI breakpoints**	**N** ** (%)**
≤ 19 mm	R	0 (0)
20–24 mm	SDD	17 (4.13)
≥ 25 mm	S	395 (95.87)
Total, *N* (%)		412 (100)

Abbreviations: R, resistant; S, susceptible, SDD, susceptible dose–dependent.

**Table 4:** Ceftaroline MIC values for MRSA clinical isolates.

**MIC (*μ*g/ml)**	**Interpretation according to CLSI breakpoints**	**Total, ** **N** ** (%)**
≥ 8 *μ*g/mL	R	0 (0.0)

2–4 *μ*g/mL	SDD	5 (1.21)
4 *μ*g/mL		2 (0.49)
2 *μ*g/mL		3 (0.73)

≤ 1 mg/L	S	407 (98.79)
1		65 (15.78)
0.5		166 (40.29)
0.25		152 (36.89)
0.125		24 (5.83)

		412 (100)

Abbreviations: R, resistant; S, susceptible; SDD, susceptible dose–dependent.

**Table 5:** Results of *spa* typing.

**Isolate ID**	***spa* type**	**Reliability**	**Reliability rate**	**F**	**R**	**Contig**	**Repeats**
145	t037	Excellent	120	259 nt.	251 nt.	206 nt.	15-12-16-02-25-17-24
154	t037	Excellent	120	258 nt.	226 nt.	206 nt.	15-12-16-02-25-17-24
190	t037	Excellent	120	477 nt.	486 nt.	206 nt.	15-12-16-02-25-17-24

Abbreviations: F, forward; R, reverse.

## Data Availability

The data presented in this study are available upon request from the corresponding author.

## References

[1] Flynn C. E., Guarner J. (2023). Emerging Antimicrobial Resistance. *Modern Pathology*.

[2] Azzam A., Khaled H., Mosa M. (2023). Epidemiology of Clinically Isolated Methicillin-Resistant *Staphylococcus aureus* (MRSA) and Its Susceptibility to Linezolid and Vancomycin in Egypt: A Systematic Review With Meta-Analysis. *BMC Infectious Diseases*.

[3] Alfeky A.-A. E., Tawfick M. M., Ashour M. S. E.-D., El-Moghazy A.-N. A. (2022). Molecular Characterization of Key Virulence Traits Among Multidrug-Resistant Methicillin-Resistant *Staphylococcus aureus* Isolates From Some Egyptian Hospitals. *Al-Azhar Journal of Pharmaceutical Sciences*.

[4] Hassan R., Barwa R., MM E. L.-S., Ashraf D. (2017). Virulence Characteristics of Methicillin-Resistant *Staphylococcus aureus* Isolated From Different Clinical Sources. *New Egyptian Journal of Microbiology*.

[5] Nour El-Din H. T., Yassin A. S., Ragab Y. M., Hashem A. M. (2021). Phenotype-Genotype Characterization and Antibiotic-Resistance Correlations Among Colonizing and Infectious Methicillin-Resistant *Staphylococcus aureus* Recovered From Intensive Care Units. *Infection and Drug Resistance*.

[6] Jevons M. P. (1961). “Celbenin” - Resistant Staphylococci. *British Medical Journal*.

[7] Moellering R. C. (2012). MRSA: The First Half Century. *Journal of Antimicrobial Chemotherapy*.

[8] Garcia-Alvarez L., Holden M. T., Lindsay H. (2011). Meticillin-Resistant *Staphylococcus aureus* With a Novel *mec*A Homologue in Human and Bovine Populations in the UK and Denmark: A Descriptive Study. *Lancet Infectious Diseases*.

[9] Paterson G. K., Larsen J., Harrison E. M. (2012). First Detection of Livestock-Associated Meticillin-Resistant *Staphylococcus aureus* CC398 in Bulk Tank Milk in the United Kingdom, January to July 2012. *Euro Surveillance*.

[10] Chang J., Tasellari A., Wagner J. L., Scheetz M. H. (2023). Contemporary Pharmacologic Treatments of MRSA for Hospitalized Adults: Rationale for Vancomycin Versus Non-Vancomycin Therapies as First Line Agents. *Expert Review of Anti-Infective Therapy*.

[11] Long S. W., Olsen R. J., Mehta S. C. (2014). PBP2a Mutations Causing High-Level Ceftaroline Resistance in Clinical Methicillin-Resistant *Staphylococcus aureus* Isolates. *Antimicrobial Agents and Chemotherapy*.

[12] Sachu A. (2023). Ceftaroline Susceptibility Among Isolates of MRSA: A Comparison of EUCAST and CLSI Breakpoints. *Ethiopian Journal of Health Sciences*.

[13] Abate G., Wang G., Frisby J. (2022). Ceftaroline: Systematic Review of Clinical Uses and Emerging Drug Resistance. *Annals of Pharmacotherapy*.

[14] Lee H., Yoon E. J., Kim D. (2018). Ceftaroline Resistance by Clone-Specific Polymorphism in Penicillin-Binding Protein 2a of Methicillin-Resistant *Staphylococcus aureus*. *Antimicrobial Agents and Chemotherapy*.

[15] Schaumburg F., Peters G., Alabi A., Becker K., Idelevich E. A. (2016). Missense Mutations of PBP2a Are Associated With Reduced Susceptibility to Ceftaroline and Ceftobiprole in African MRSA. *Journal of Antimicrobial Chemotherapy*.

[16] Ramadan A. A. (2022). Bacterial Typing Methods From Past to Present: A Comprehensive Overview. *Gene Reports*.

[17] Mollerup S., Worning P., Petersen A., Bartels M. D. (2022). spa Typing of Methicillin-Resistant *Staphylococcus aureus* Based on Whole-Genome Sequencing: The Impact of the Assembler. *Microbiology Spectrum*.

[18] CLSI (2023). *Performance Standards for Antimicrobial Susceptibility Testing*.

[19] Louie L., Matsumura S., Choi E., Louie M., Simor A. (2000). Evaluation of Three Rapid Methods for Detection of Methicillin Resistance in *Staphylococcus aureus*. *Journal of Clinical Microbiology*.

[20] Murakami K., Minamide W., Wada K., Nakamura E., Teraoka H., Watanabe S. (1991). Identification of Methicillin-Resistant Strains of Staphylococci by Polymerase Chain Reaction. *Journal of Clinical Microbiology*.

[21] Deplano A., Vandendriessche S., Nonhoff C., Denis O. (2014). Genetic Diversity Among Methicillin-Resistant *Staphylococcus aureus* Isolates Carrying the *mec*C Gene in Belgium. *The Journal of Antimicrobial Chemotherapy*.

[22] Brakstad O. G., Aasbakk K., Maeland J. A. (1992). Detection of *Staphylococcus aureus* by Polymerase Chain Reaction Amplification of the nuc Gene. *Journal of Clinical Microbiology*.

[23] Nethercott C., Mabbett A. N., Totsika M. (2013). Molecular Characterization of Endocarditis-Associated *Staphylococcus aureus*. *Journal of Clinical Microbiology*.

[24] Harmsen D., Claus H., Witte W. (2003). Typing of Methicillin-Resistant *Staphylococcus aureus* in a University Hospital Setting by Using Novel Software for spa Repeat Determination and Database Management. *Journal of Clinical Microbiology*.

[25] Mellmann A., Weniger T., Berssenbrügge C. (2007). Based Upon Repeat Pattern (BURP): An Algorithm to Characterize the Long-Term Evolution of *Staphylococcus aureus* Populations Based on *spa* Polymorphisms. *BMC Microbiology*.

[26] Sahebnasagh R., Saderi H., Owlia P. (2014). The Prevalence of Resistance to Methicillin in *Staphylococcus aureus* Strains Isolated From Patients by PCR Method for Detec-tion of *mec*A and *nuc* Genes. *Iranian Journal of Public Health*.

[27] Adeiza S. S., Onaolapo J. A., Olayinka B. O. (2020). Prevalence, risk-factors, and Antimicrobial Susceptibility Profile of Methicillin-Resistant *Staphylococcus aureus* (MRSA) Obtained From Nares of Patients and Staff of Sokoto State-Owned Hospitals in Nigeria. *GMS Hygiene and Infection Control*.

[28] Diekema D. J., Pfaller M. A., Shortridge D., Zervos M., Jones R. N. (2019). Twenty-Year Trends in Antimicrobial Susceptibilities Among *Staphylococcus aureus* From the SENTRY Antimicrobial Surveillance Program. *Open Forum Infectious Diseases*.

[29] Antimicrobial Resistance Collaborators (2022). Global Burden of Bacterial Antimicrobial Resistance in 2019: A Systematic Analysis. *Lancet*.

[30] Nourollahpour Shiadeh M., Sepidarkish M., Mollalo A. (2022). Worldwide Prevalence of Maternal Methicillin-Resistant *Staphylococcus aureus* Colonization: A Systematic Review and Meta-Analysis. *Microbial Pathogenesis*.

[31] Kaye K. S., Udeani G., Cole P., Friedland H. D. (2015). Ceftaroline Fosamil for the Treatment of Hospital-Acquired Pneumonia and Ventilator-Associated Pneumonia. *Hospital Practice*.

[32] Pasquale T. R., Tan M. J., Trienski T. L., File T. M. (2015). Methicillin-Resistant *Staphylococcus aureus* Nosocomial Pneumonia Patients Treated With Ceftaroline: Retrospective Case Series of 10 Patients. *Journal of Chemotherapy*.

[33] Alfeky A. E., Tawfick M. M., Ashour M. S., El-Moghazy A. A. (2022). High Prevalence of Multi-Drug Resistant Methicillin-Resistant *Staphylococcus aureus* in Tertiary Egyptian Hospitals. *Journal of Infection in Developing Countries*.

[34] Falagas M. E., Karageorgopoulos D. E., Leptidis J., Korbila I. P. (2013). MRSA in Africa: Filling the Global Map of Antimicrobial Resistance. *PLoS One*.

[35] Refeai S. A., Kamal N. N., Ghazawy E. R. A., Fekry C. M. (2020). Perception and Barriers Regarding Infection Control Measures Among Healthcare Workers in Minia City, Egypt. *International Journal of Preventive Medicine*.

[36] Salem M. R., Youssef M. R. L. (2017). Health Care Providers’ Perspectives for Providing Quality Infection Control Measures at the Neonatal Intensive Care Unit, Cairo University Hospital. *American Journal of Infection Control*.

[37] El-Hawy R. M., Ashmawy M. I., Kamal M. M. (2017). Studying the Knowledge, Attitude and Practice of Antibiotic Misuse Among Alexandria Population. *European Journal of Hospital Pharmacy*.

[38] Elsayed A. A., Darwish S. F., Zewail M. B., Mohammed M., Saeed H., Rabea H. (2021). Antibiotic Misuse and Compliance With Infection Control Measures During COVID-19 Pandemic in Community Pharmacies in Egypt. *International Journal of Clinical Practice*.

[39] Maarouf L., Amin M., Evans B. A., Abouelfetouh A. (2023). Knowledge, Attitudes and Behaviour of Egyptians Towards Antibiotic Use in the Community: Can We Do Better?. *Antimicrobial Resistance and Infection Control*.

[40] Alfouzan W., Boswihi S. S., Dhar R., Udo E. (2020). Evaluating the Antibacterial Activity of Ceftaroline Against Clinical Isolates of Methicillin-Susceptible and- Resistant *Staphylococcus aureus* in Kuwait Hospitals. *Journal of Infection and Public Health*.

[41] Monecke S., Bedewy A. K., Muller E. (2023). Characterisation of Methicillin-Resistant *Staphylococcus aureus* From Alexandria, Egypt. *Antibiotics*.

[42] Abdelwahab M. A., Amer W. H., Elsharawy D. (2023). Phenotypic and Genotypic Characterization of Methicillin Resistance in Staphylococci Isolated From an Egyptian University Hospital. *Pathogens*.

[43] Shebl H., Zaki W., Saleh A., Salam S. J. J. P. A. M. (2020). Prevalence of MecC Gene Among Methicillin Resistant *Staphylococcus aureus* Isolated From Patients in Ain-Shams University Hospital. *Journal of Pure and Applied Microbiology*.

[44] Girgis S. A., Gomaa H. E., Saad N. E., Salem M. M. J. L. S. J. (2013). A Comparative Study for Detection of Methicillin Resistance *Staphylococci* by Polymerase Chain Reaction and Phenotypic Methods. *Life Science Journal*.

[45] Khan A. A., Ali A., Tharmalingam N., Mylonakis E., Zahra R. (2020). First Report of *mec*C Gene in Clinical Methicillin Resistant *S. aureus* (MRSA) From Tertiary Care Hospital Islamabad, Pakistan. *Journal of Infection and Public Health*.

[46] Al-Zoubi M. S., Al-Tayyar I. A., Hussein E., Jabali A. A., Khudairat S. (2015). Antimicrobial Susceptibility Pattern of *Staphylococcus aureus* Isolated From Clinical Specimens in Northern Area of Jordan. *Iranian Journal of Microbiology*.

[47] Assefa M. (2022). Inducible Clindamycin-Resistant *Staphylococcus aureus* Strains in Africa: A Systematic Review. *International Journal of Microbiology*.

[48] Chang V. S., Dhaliwal D. K., Raju L., Kowalski R. P. (2015). Antibiotic Resistance in the Treatment of *Staphylococcus aureus* Keratitis: A 20-Year Review. *Cornea*.

[49] Entenza J. M., Que Y. A., Vouillamoz J., Glauser M. P., Moreillon P. (2001). Efficacies of Moxifloxacin, Ciprofloxacin, and Vancomycin Against Experimental Endocarditis due to Methicillin-Resistant *Staphylococcus aureus* Expressing Various Degrees of Ciprofloxacin Resistance. *Antimicrobial Agents and Chemotherapy*.

[50] Lemaire S., Kosowska-Shick K., Appelbaum P. C., Glupczynski Y., Van Bambeke F., Tulkens P. M. (2011). Activity of Moxifloxacin Against Intracellular Community-Acquired Methicillin-Resistant *Staphylococcus aureus*: Comparison With Clindamycin, Linezolid and Co-Trimoxazole and Attempt at Defining an Intracellular Susceptibility Breakpoint. *Journal of Antimicrobial Chemotherapy*.

[51] Vola M. E., Moriyama A. S., Lisboa R. (2013). Prevalence and Antibiotic Susceptibility of Methicillin-Resistant *Staphylococcus aureus* in Ocular Infections. *Arquivos Brasileiros de Oftalmologia*.

[52] Alseqely M., Newton-Foot M., Khalil A., El-Nakeeb M., Whitelaw A., Abouelfetouh A. (2021). Association Between Fluoroquinolone Resistance and MRSA Genotype in Alexandria, Egypt. *Scientific Reports*.

[53] El-Sokkary R. H., Ramadan R. A., El-Shabrawy M. (2018). Community Acquired Pneumonia Among Adult Patients at an Egyptian University Hospital: Bacterial Etiology, Susceptibility Profile and Evaluation of the Response to Initial Empiric Antibiotic Therapy. *Infection and Drug Resistance*.

[54] Agmy G., Mohamed S., Gad Y., Farghally E., Mohammedin H., Rashed H. (2013). Bacterial Profile, Antibiotic Sensitivity and Resistance of Lower Respiratory Tract Infections in Upper Egypt. *Mediterranean Journal of Hematology and Infectious Diseases*.

[55] Ashour H. M., el-Sharif A. (2007). Microbial Spectrum and Antibiotic Susceptibility Profile of Gram-Positive Aerobic Bacteria Isolated From Cancer Patients. *Journal of Clinical Oncology*.

[56] Zhang Z., Chen M., Yu Y., Liu B., Liu Y. (2019). In Vitro Activity of Ceftaroline and Comparators Against *Staphylococcus aureus* Isolates: Results From 6 Years of the ATLAS Program (2012 to 2017). *Infection and Drug Resistance*.

[57] Berends E. T., Horswill A. R., Haste N. M., Monestier M., Nizet V., von Kockritz-Blickwede M. (2010). Nuclease Expression by *Staphylococcus aureus* Facilitates Escape From Neutrophil Extracellular Traps. *Journal of Innate Immunity*.

[58] Kiedrowski M. R., Kavanaugh J. S., Malone C. L. (2011). Nuclease Modulates Biofilm Formation in Community-Associated Methicillin-Resistant *Staphylococcus aureus*. *PLoS One*.

[59] Mann E. E., Rice K. C., Boles B. R. (2009). Modulation of eDNA Release and Degradation Affects *Staphylococcus aureus* Biofilm Maturation. *PLoS One*.

[60] Hoegh S. V., Skov M. N., Boye K., Worning P., Jensen T. G., Kemp M. (2014). Variations in the *Staphylococcus aureus*-Specific *nuc* Gene Can Potentially Lead to Misidentification of Meticillin-Susceptible and -Resistant S. aureus. *Journal of Medical Microbiology*.

[61] van Leeuwen W., Roorda L., Hendriks W., Francois P., Schrenzel J. (2008). Anuc-Deficient Meticillin-Resistant Staphylococcus aureusstrain. *FEMS Immunology & Medical Microbiology*.

[62] Rania A. A., Nsreen M. K., HEl R., Mona M. A. (2017). Evaluation for the Novel mecC Methicillin Resistance Among Methicillin Resistant Staphylococcal Isolates in Two Egyptian University Hospitals. *Archives of Clinical Microbiology*.

[63] Al-Dulaimi T., Al-Marzoqi A. H., Ahmed N. K. (2014). Phenotypic Detection of Resistance in *Staphylococcus aureus* Isolates: Detection of (*mec* A and *fem* A) Gene in Methicillin Resistant *Staphylococcus aureus* (MRSA) by Polymerase Chain Reaction. *Journal of Natural Sciences Research*.

[64] Dhungel S., Rijal K. R., Yadav B. (2021). Methicillin-Resistant *Staphylococcus aureus* (MRSA): Prevalence, Antimicrobial Susceptibility Pattern, and Detection of *mec*A Gene Among Cardiac Patients From a Tertiary Care Heart Center in Kathmandu, Nepal. *Infectious Diseases*.

[65] Ba X., Harrison E. M., Edwards G. F. (2014). Novel Mutations in Penicillin-Binding Protein Genes in Clinical *Staphylococcus aureus* Isolates That Are Methicillin Resistant on Susceptibility Testing, but Lack the *mec* Gene. *The Journal of Antimicrobial Chemotherapy*.

[66] Garcia Alvarez L., Webb C. R., Holmes M. A. (2011). A Novel Field-Based Approach to Validate the Use of Network Models for Disease Spread Between Dairy Herds. *Epidemiology and Infection*.

[67] Becker K., Ballhausen B., Köck R., Kriegeskorte A. (2014). Methicillin Resistance in *Staphylococcus* Isolates: The “*mec* Alphabet” With Specific Consideration of *mec*C, a *mec* Homolog Associated With Zoonotic *S. aureus* Lineages. *International Journal of Medical Microbiology*.

[68] Becker K., Denis O., Roisin S. (2016). Detection of *mec*A- and *mec*C-Positive Methicillin-Resistant *Staphylococcus aureus* (MRSA) Isolates by the New Xpert MRSA Gen 3 PCR Assay. *Journal of Clinical Microbiology*.

[69] Cassone M., Mantey J., Gontjes K. J. (2021). Seasonal Patterns in Incidence and Antimicrobial Resistance of Common Bacterial Pathogens in Nursing Home Patients and Their Rooms. *Frontiers in Public Health*.

[70] Ciesielczuk H., Xenophontos M., Lambourne J. (2019). Methicillin-Resistant *Staphylococcus aureus* Harboring *mec*C Still Eludes Us in East London, United Kingdom. *Journal of Clinical Microbiology*.

[71] Lozano C., Fernandez-Fernandez R., Ruiz-Ripa L., Gomez P., Zarazaga M., Torres C. (2020). Human *mec*C-Carrying MRSA: Clinical Implications and Risk Factors. *Microorganisms*.

[72] Paterson G. K., Harrison E. M., Holmes M. A. (2014). The Emergence of *mec*C Methicillin-Resistant *Staphylococcus aureus*. *Trends in Microbiology*.

[73] Petersen A., Stegger M., Heltberg O. (2013). Epidemiology of Methicillin-Resistant *Staphylococcus aureus* Carrying the Novel *mec*C gene in Denmark Corroborates a Zoonotic Reservoir With Transmission to Humans. *Clinical Microbiology and Infection*.

[74] Shore A. C., Deasy E. C., Slickers P. (2011). Detection of Staphylococcal Cassette Chromosome mec Type XI Carrying Highly Divergent *mec*A, *mec*I, *mec*R1, *bla*Z, and *ccr* Genes in Human Clinical Isolates of Clonal Complex 130 Methicillin-Resistant *Staphylococcus aureus*. *Antimicrobial Agents and Chemotherapy*.

[75] Stegger M., Andersen P. S., Kearns A. (2012). Rapid Detection, Differentiation and Typing of Methicillin-Resistant *Staphylococcus aureus* Harbouring Either *mec*A or the New *mec*A Homologue *mec*A(LGA251). *Clinical Microbiology and Infection*.

[76] Cikman A., Aydin M., Gulhan B. (2019). Absence of the *mec*C Gene in Methicillin-Resistant *Staphylococcus aureus* Isolated From Various Clinical Samples: The First Multi-Centered Study in Turkey. *Journal of Infection and Public Health*.

[77] Kilic A., Dogan E., Kaya S., Baysallar M. (2015). Investigation of the Presence of *mec*C and Panton-Valentine Leukocidin Genes in *Staphylococcus aureus* Strains Isolated From Clinical Specimens During Seven Years Period. *Mikrobiyoloji Bülteni*.

[78] Nijjar C. K., Smith M. H., Eltringham I. J. (2014). Adjunctive *mec*A PCR for Routine Detection of Methicillin Susceptibility in Clinical Isolates of Coagulase-Negative Staphylococci. *Journal of Clinical Microbiology*.

[79] Peterson J. C., Durkee H., Miller D. (2019). Molecular Epidemiology and Resistance Profiles Among Healthcare- and Community-Associated *Staphylococcus aureus* Keratitis Isolates. *Infection and Drug Resistance*.

[80] Khairalla A. S., Wasfi R., Ashour H. M. (2017). Carriage Frequency, Phenotypic, and Genotypic Characteristics of Methicillin-Resistant _Staphylococcus aureus_ Isolated From Dental Health-Care Personnel, Patients, and Environment. *Scientific Reports*.

[81] Banerjee R., Gretes M., Harlem C., Basuino L., Chambers H. F. (2010). AmecA-Negative Strain of Methicillin-Resistant Staphylococcus aureus With High-Level *β*-Lactam Resistance Contains Mutations in Three Genes. *Antimicrobial Agents and Chemotherapy*.

[82] Hryniewicz M. M., Garbacz K. (2017). Borderline Oxacillin-Resistant *Staphylococcus aureus* (BORSA) - A More Common Problem Than Expected?. *Journal of Medical Microbiology*.

[83] Barg N., Chambers H., Kernodle D. (1991). Borderline Susceptibility to Antistaphylococcal Penicillins Is not Conferred Exclusively by the Hyperproduction of Beta-Lactamase. *Antimicrobial Agents and Chemotherapy*.

[84] Asadollahi P., Farahani N. N., Mirzaii M. (2018). Distribution of the Most Prevalent Spa Types Among Clinical Isolates of Methicillin-Resistant and -Susceptible *Staphylococcus aureus* Around the World: A Review. *Frontiers in Microbiology*.

[85] Aiken A. M., Mutuku I. M., Sabat A. J. (2014). Carriage of *Staphylococcus aureus* in Thika Level 5 Hospital, Kenya: A Cross-Sectional Study. *Antimicrobial Resistance and Infection Control*.

[86] Singh-Moodley A., Lowe M., Mogokotleng R., Perovic O. (2020). Diversity of SCC*mec* Elements and *spa* Types in South African *Staphylococcus aureus mec*A-Positive Blood Culture Isolates. *BMC Infectious Diseases*.

